# The Cholera

**Published:** 1848-01

**Authors:** 


### THE CHOLERA.

Official accounts from St. Petersburg, dated the 12th Nov., announce that the cholera had made fresh progress at Moscow. Between the 25th of October and 1st of November the number of cases daily increased, 641 persons having been attacked during that period, 238 of whom had died. From the first appearance of the malady in that city up to the 1st of November, 1197 cases occurred, 402 of which proved fatal. The patients belonged for the most part to the lower classes. The cholera had totally ceased in the government of Astrakan, where it carried off 3772 persons, and in that of Koursk, where 1087 died out of 1673 patients. At Kazan there were 1224 cases and 665 deaths. At Kleff the cholera was likewise increasing in intensity. Since the 26th of October, 278 were attacked and 113 died.

